# Effect of Naringin Treatment on Postmenopausal Osteoporosis in Ovariectomized Rats: A Meta-Analysis and Systematic Review

**DOI:** 10.1155/2021/6016874

**Published:** 2021-02-10

**Authors:** Zhu Zhu, Wenjing Xie, Yanyan Li, Zaiou Zhu, Wei Zhang

**Affiliations:** ^1^Jiangsu Key Laboratory of Oral Diseases, Nanjing Medical University, Nanjing, Jiangsu, China; ^2^Department of Oral Special Consultation, Affiliated Stomatological Hospital of Nanjing Medical University, Nanjing, Jiangsu, China; ^3^Department of Oral and Maxillofacial Surgery, Affiliated Stomatological Hospital of Nanjing Medical University, Nanjing, Jiangsu, China

## Abstract

**Background:**

Osteoporosis is a major disease that affects the quality of life of middle-aged and old people, so it is very important to find efficient and safe drugs to treat osteoporosis. The purpose of this study was to investigate the therapeutic effect of naringin on postmenopausal osteoporosis in ovariectomized (OVX) rats.

**Methods:**

Chinese biomedical databases, CNKI, PubMed, EMBASE, and Wan Fang were searched for articles from inception to March 2020. Two independent researchers screened articles according to inclusion criteria. RevMan 5.3 was used for data analysis.

**Results:**

Ten studies were included in the systematic review. The bone mineral density (BMD) significantly increased after naringin treatment (weighted mean difference, 0.06; 95% CI, 0.03–0.09; *P* < 0.01). There was no significant increase in BMD after estrogen treatment compared with naringin (weighted mean difference, 0.00; 95% CI, −0.00 to 0.01; *P* = 0.06). The trabecular bone volume (BV/TV) (weighted mean difference, 2.09; 95% CI, 1.85–2.34; *P* < 0.01) and trabecular thickness (Tb.Th) (weighted mean difference, 6.65; 95% CI, 6.55–6.74; *P* < 0.01) significantly increased after using naringin.

**Conclusions:**

Naringin had been shown to promote bone formation in OVX rats. However, the mechanism of naringin needs more research to confirm.

## 1. Background

Osteoporosis is a global public health problem, which primarily threatens postmenopausal women and senior citizens [1]. The pathological characteristics of osteoporosis include bone mineral density (BMD) reduction, bone microstructure deterioration, and matrix protein degradation, resulting in an increased bone fragility and risk of fracture [2]. It not only reduces the quality of life of the elderly, but also causes huge financial burden to patients' families and society. Postmenopausal osteoporosis is the most common type of osteoporosis [3]. At menopause, estrogen withdrawal accelerates bone remodeling with a net increase in bone resorption, which leads to bone loss and even osteoporosis [4, 5]. The ovariectomized (OVX) rats are the most commonly used animal models to study postmenopausal osteoporosis. In fact, so far, a fully curative treatment for osteoporosis has yet to be developed. In an effort to discover new drugs that treat osteoporosis effectively, much focus has been put on the pursuit of natural-based products because of their availability, cost-effectiveness, and biological activity.

Naringin, a polymethoxylated flavonoid glycoside, is the main bioactive flavonoid extracted from citrus fruits [6, 7] and has a positive effect on osteoporosis caused by postmenopause, glucocorticoid, orchidectomy, and aging [8]. Studies have shown that naringin can promote the differentiation and proliferation of various types of cells [9–12] and improve the bone mass in an osteoporotic rat model [9]. Naringin also promotes osteoclast apoptosis through the mitochondrial-mediated apoptotic pathway, so as to inhibit bone loss in OVX rat models and exhibit antiosteoporotic pharmacological activity [13, 14].

The main purpose of this systematic review was to collect and analyze all the data available to investigate the therapeutic effect of naringin on postmenopausal osteoporosis in OVX rats.

## 2. Methods

### 2.1. Selection of Studies

All articles published in Chinese and English databases from their inception to March 2020 were searched, including Chinese Biomedical databases, CNKI, PubMed, EMBASE, and Wan Fang. Search keywords were as follows: ovariectomized, ovariectomy, ovariectomies, bone diseases, osteoporosis, bone loss, bone mineral density, naringin (And or OR). Meanwhile, the references of selected articles were used as a supplement. There were no language restrictions. This review followed the Preferred Reporting Items for Systematic Reviews and Meta-Analyses (PRISMA) reporting criteria [15]. The Stroke Therapy Academic Industry Roundtable (STAIR) list was used for methodological quality assessment in our study [16].

### 2.2. Inclusion and Exclusion Criteria

Studies were included if they met all of the following criteria: studies with (1) experimental and control groups; (2) OVX-induced bone loss and osteoporosis; (3) evaluation of therapeutic effects of naringin. Exclusion criteria were as follows: (1) duplicate articles; (2) studies without control groups; (3) studies without the use of BMD; (4) all clinical case reports and only in vitro studies.

### 2.3. Data Extraction

According to inclusion criteria, two investigators (Z. Z. and W. X.) independently scrutinized all articles and the reference list of all relevant articles was also screened to identify other potential data sources. Data were also extracted from these studies independently by two investigators (Z. Z. and W. X.) using a spreadsheet, including column names of the first author, publication date, the age and species of animal studies, experimental designs, naringin treatment dosage, administration time, and intervention protocols. If two authors disagreed on the choice of research and data interpretation, inconsistencies were settled by discussion with another independent investigator (Y. L.). The final results needed to be discussed with all the investigators to reach a consensus.

### 2.4. Statistical Analysis

Statistical analyses were performed using RevMan 5.3 (Cochrane Collaboration, Oxford, United Kingdom). Heterogeneity among studies was assessed using the *I*^2^ statistic [17]. I^2^ <50% meant that the homogeneity of the study was high and there was no significant heterogeneity, so the fixed effect model should be used. Otherwise, the random effect model was used. *P* < 0.05 indicated significant differences between groups. Continuous variable data were analyzed with the relative risk or weighted mean difference and its 95% confidence interval (CI).

## 3. Results

### 3.1. Selection of Studies

**Figure 1:**
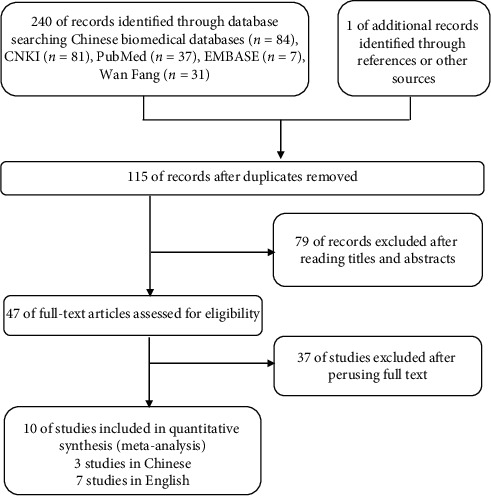
Flow diagram of the article selection process for review of Chinese biomedical databases, CNKI, PubMed, EMBASE, and Wan Fang.

A total of 241 related studies were selected by searching the databases and the literature references of the articles retrieved. After deleting duplicates, 47 studies remained by reading their titles and abstracts. A thorough review of the remaining 47 articles was carried out, of which 37 articles were excluded. The reason for exclusion was the absence of BMD measurements, being clinic reports, or studies only in vitro. Ultimately, 10 studies (three studies were published in Chinese and seven studies were published in English) were selected [8, 9, 18–25] for this systematic review. The flow diagram of the study selection process is shown in Figure 1.

### 3.2. Characteristics of the Included Studies

Characteristics of the studies are shown in Table 1. Eight studies used Sprague-Dawley (80%) female rats, one study [18] used C57/BL6j (10%) female rats, and another study [21] did not specify which animal model to use. The age range of OVX in rats was 1∼6 weeks, except for one study which did not report the age of the rats. In a total of 10 studies, the animal sample size ranged from 20 to 75, and the median sample size was 48 rats. All the studies were administered by oral gavage. The daily dosage range of naringin was 1–1500 mg/kg. The duration of naringin administration varied from 2 to 13 weeks. In all studies, BMD was used to evaluate the results. 3 studies provided data on trabecular bone volume (BV/TV) and trabecular thickness (Tb.Th).

### 3.3. Risk of Bias

In general, the methodological quality of the studies was not high. None of the studies included any description of sample size calculation or inclusion and exclusion criteria. All studies had reported randomization; however, none of them described allocation concealment. Further, none of the studies reported on their sources of funding or support or any potential conflicts of interest.  Table 2 shows the risk of bias reported for each publication included in this meta-analysis.

### 3.4. BMD BV/TV Tb.Th Changes after Using Naringin

**Figure 2:**
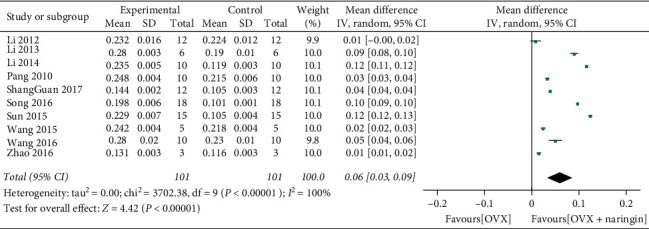
BMD changes after naringin treatment.

**Figure 3:**
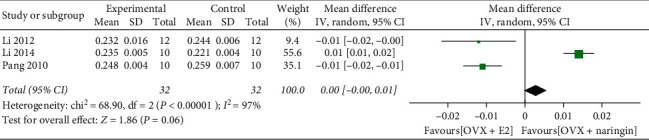
Comparison of the effects of naringin and estrogen on BMD.

Due to the high heterogeneity of the study, we adopted the random effect model. Compared with the blank control group, the BMD significantly increased after naringin treatment (ten studies, *n* = 101; weighted mean difference, 0.06; 95% CI, 0.03–0.09; *P* < 0.01) (Figure 2). Besides, in studies where estrogen treatment served as the positive control group, we found that there was no significant increase in BMD after estrogen treatment compared with naringin (three studies, *n* = 32; weighted mean difference, 0.00; 95% CI, −0.00 to 0.01; *P* = 0.06) (Figure 3).

**Figure 4:**
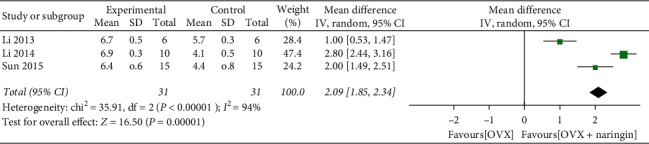
BV/TV changes after naringin treatment.

**Figure 5:**
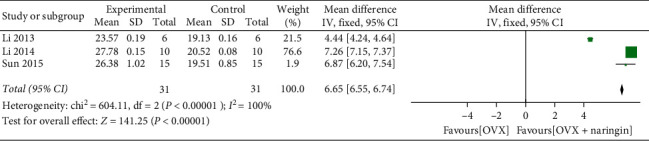
Tb.Th changes after naringin treatment.

Only three studies [8, 21, 22] used BV/TV, Tb.Th as outcome measures. The BV/TV (%) values significantly increased after using naringin (three studies, *n* = 31; weighted mean difference, 2.09; 95% CI, 1.85–2.34; *P* < 0.01) (Figure 4). There was a statistically significant difference in Tb.Th (um) values between the two groups. The weighted mean difference was found to be 6.65, with 95% CI = 6.55–6.74 and *P* < 0.01 (Figure 5).

## 4. Discussion

Osteoporosis is an age-related disease that affects millions of people. Although the application of traditional Chinese medicine in the treatment of osteoporosis has a long history, its specific mechanism is not known yet [26]. The research on the monomer of traditional Chinese medicine still remains in basic research. For this, we focus our attention on animal models.

OVX is mainly used to establish animal models of osteoporosis [27]. OVX reduces the level of estrogen and induces bone loss in animals, which is similar to postmenopausal bone loss [28], including rapid bone loss and bone resorption, as well as similar bone reactions to therapy with estrogen, bisphosphonates, tamoxifen, and calcitonin. These wide-ranging similarities make OVX animal models widely used as clinically relevant models of postmenopausal bone loss in women [29]. The OVX rat model was approved by the US Food and Drug Administration (FDA) as a preclinical model [30]. Systematic reviews of animal experiments can improve the accuracy of clinical trial effectiveness prediction, reduce the risk of negative results, and also determine when clinical results of animal experiments can be accepted and terminate unnecessary clinical trials.

As far as we know, this is the first meta-analysis to report the effect of naringin on bone mass in OVX rats. We used random effect models to study. The results showed that the BMD value of the experimental group increased significantly, indicating that naringin could promote bone formation in OVX rats and improve osteoporosis caused by estrogen deficiency. In addition, the effect of naringin in OVX rats was not significantly different from that of estrogen, suggesting that the mechanism of naringin may be the same as that of estrogen, giving us a new direction of research. Compared with the control group, the BV/TV and Tb.Th values of naringin treated group were significantly higher, which also proved the effect of naringin on bone formation.

BMD is an important sign of bone quality, which is of great significance in medicine [31]. It is of great significance in the occurrence, diagnosis, treatment, prognosis, and follow-up observation of osteoporosis, osteomalacia, fibrosis, and other diseases that affect calcium and phosphorus metabolism [32]. BMD was calculated by x-ray absorption of bone. In all ten studies, most of the studies were conducted with dual energy x-ray absorptiometry (DEXA).

With the help of image processing software, regions of interest (ROI) can be selected on the scanning image of micro-CT for threshold segmentation, so that cortical bone and cancellous bone can be segmented and extracted into different tissue regions, respectively [33]. Finally, various morphological characteristics of cortical bone and cancellous bone (trabecular bone) can be studied and analyzed. In general, when the bone tissue in the ROI is the same, the BV/TV value can reflect the bone mass, which is a common index for evaluating the bone mass of cortical bone and cancellous bone [34]. For the cancellous bone in the medullary cavity, the BV/TV value can reflect the trabecular bone mass of different samples. The increased value indicates that bone metabolism is greater than catabolism and bone mass increases, thereby indirectly reflecting bone metabolism. It has the same value in evaluating bone mass and bone metabolism in the middle part of long bone.

The porous trabecular structure connected with trabecular bone is regularly arranged in accordance with the stress curve and has nonuniform anisotropy. This arrangement can increase the bone strength [35]. It can be said that the bone mass of the bone trabeculae is closely related to its microstructure. Therefore, the microstructural analysis of trabecular bone is very important in bone analysis. Micro-CT can perform nondestructive 3D imaging of trabecular bone microstructure and display the microstructure of trabecular bone, making it possible to analyze the topology of trabecular bone microstructure [36]. Tb.Th is one of the main indexes to evaluate the spatial structure of trabeculae [37]. When bone catabolism is greater than bone anabolism, such as in the case of osteoporosis, the Tb.Th value decreases, whereas it increases when bone anabolism is greater than bone catabolism.

Functions of naringin in bone development are largely unknown. Many studies have reported the potential mechanism of naringin promoting osteogenesis. Wang et al. observed that coadministration of AMPK and Akt inhibitors partly reversed naringin effects in vivo, suggesting that the osteogenic activity of this flavonoid is in part via its stimulation of the Wnt/b-catenin signaling upon interaction with AMPK and Akt [20]. Studies found that naringin inhibits osteoclast formation and bone resorption by the suppression of RANKL-induced activation of NF-kB and ERK [38]. Fan et al. revealed that naringin is able to promote BMSC differentiation into osteoblasts, via the upregulation of miR-20a, and the downregulation of PPAR*γ* [39]. Wu et al. demonstrated that naringin increased BMP-2 expression via PI3K, Akt, c-Fos/c-Jun, and AP-1-dependent pathways, which can induce osteoblast proliferation, differentiation, and maturation in cultured osteoblasts [40]. In this study, we hypothesized that the mechanism of naringin should be similar to estrogen. Naringin exerted antiosteoporotic effects by binding to estrogen receptors (ERs), which may replace the estrogen replacement therapy (ERT) in clinical use [41], but more experiments were needed to confirm this hypothesis [42].

The STAIR list was used in this systematic review, which was concise and contained the basic criteria that focus on high quality, such as sample size calculation, randomization, allocation concealment, and blinded outcome assessment.

This meta-analysis also had many limitations. First, despite the best efforts we had made, there was no way to ensure that all relevant studies were found. Second, the quality evaluation of this research is low. There was a lack of calculation of sample size, explanation of random allocation method, and reports of animals excluded from analysis. At the same time, there were biases caused by publication bias. In particular, systematic reviews of animal studies were more susceptible to publication bias than clinical studies [43]. Third, all results (BMD, BV/TV, and Tb. Th) were highly heterogeneous in this study, indicating that the results may not be convincing. For BV/TV and Tb.Th index, it was difficult to establish a unified ROI, which resulted in a large heterogeneity between the studies. The different time of OVX and the different dosage and durations of naringin treatment in rats may lead to higher heterogeneity. However, due to the small number of studies, there was limited ability to consider subgroup analyses. Fourth, this study did not accurately infer the optimal concentration of naringin in OVX rats and could not provide guidance for future animal experiments, so more research and analysis are needed.

## 5. Conclusions

In OVX rats model, the BMD, BV/TV, and Tb. Th values of naringin treatment group were significantly increased, which proved that naringin promoted bone formation. At the same time, we speculated that the mechanism of naringin was the same as estrogen, which needs more research to confirm.

## Figures and Tables

**Table 1:** Characteristics of the included studies.

Study	Animals	Age, mo	Sample size	Experimental group	Control group	Duration, wk
Pang et al 2010	C57/BL6J mice	1	10/10/10/10/10	A : OVX + naringin (200 mg/kg daily); B : OVX + naringin (400 mg/kg daily)	A: sham vehicle (2% ethanol); B : OVX + vehicle; C : OVX + 17b-estradiol (E2, 2 ug/g daily)	6
Shangguan et al. 2017	SD rats (body weight, 220 ± 8 g)	6	12/12/12/12	A : OVX + naringin (200 mg/kg daily); B : OVX + naringin (100 mg/kg daily)	A: sham group (physiological saline solution, 6 ml/kg daily); B : OVX group (physiological saline solution, 6 ml/kg daily)	12
Li et al. 2014	SD rats (body weight, 230 ± 10 g)	6	10/10/10/10/10/10	A : OVX + naringin (40 mg/kg daily); B : OVX + naringin (100 mg/kg daily); C : OVX + naringin (200 mg/kg daily)	A: sham group (H2O); B : OVX group (H2O); C : OVX + E2 (22.5 ug/kg daily)	12
Song et al. 2016	SD rats (body weight, 240 ± 12 g)	3	18/18/18/18	A : OVX + naringin (40 mg/kg daily); B : OVX + naringin (100 mg/kg daily); C : OVX + naringin (300 mg/kg daily)	B : OVX group (normal saline)	8
Wang et al. 2015	Unclear	1	5/5/5/5	A : OVX naringin (5 nM); B : OVX naringin with PTH	A: sham vehicle; B : OVX vehicle (PBS)	6
Sun et al. 2015	SD rats (body weight, 230 ± 10 g)	3	15/15/15/15/15	A : OVX naringin (300 mg/kg daily); B : OVX naringin plus treadmill exercise (300 mg/kg daily + EX)	A: sham group (H2O); B : OVX vehicle; C : OVX exercise	12
Li et al. 2013	SD rats (body weight, 220g)	3	6/6/6/6	A : OVX + naringin (60 mg/kg daily); B : OVX + naringin (300 mg/kg daily); C : OVX + naringin (1500 mg/kg daily)	A : OVX vehicle (PBS)	8
Li et al. 2012	SD rats (body weight, 280g)	4	12/12/12/12/12	A : OVX + naringin (4 mg/kg daily); B : OVX + naringin (2 mg/kg daily)	A: sham group (H2O); B : OVX vehicle (H2O); C : OVX + E2	13
Wang et al. 2016	SD rats (body weight, 230 ± 25 g)	6	10/10/10/10/10	A : OVX + naringin (1 mg/kg daily); B : OVX + naringin (10 mg/kg daily); C : OVX + naringin (100 mg/kg daily)	A: sham group; B : OVX + N	4
Zhao et al. 2016	SD rats (body weight, 230 ± 20 g)	Unclear	15/15	A : OVX + naringin (300 mg/kg daily)	A : OVX vehicle (normal saline)	2

**Table 2:** Risk of bias.

Study	Sample size calculation	Inclusion and exclusion criteria	Randomization	Allocation concealment	Reporting of animals excluded from analysis	Blinded assessment of outcome	Reporting potential conflicts of interest
Pang et al 2010	Unclear	Unclear	Yes	Unclear	Unclear	Yes	No
Shangguan et al. 2017	Unclear	Unclear	Yes	Unclear	Unclear	Yes	No
Li et al. 2014	Unclear	Unclear	Yes	Unclear	Unclear	Yes	No
Song et al. 2016	Unclear	Unclear	Yes	Unclear	Unclear	Yes	No
Wang et al. 2015	Unclear	Unclear	Yes	Unclear	Unclear	Yes	No
Sun et al. 2015	Unclear	Unclear	Yes	Unclear	Unclear	Yes	No
Li et al. 2013	Unclear	Unclear	Yes	Unclear	Unclear	Yes	No
Li et al. 2012	Unclear	Unclear	Yes	Unclear	Unclear	Yes	No
Wang et al. 2016	Unclear	Unclear	Yes	Unclear	Unclear	Yes	No
Zhao et al. 2016	Unclear	Unclear	Yes	Unclear	Unclear	Yes	No

## Data Availability

The data used to support the findings of this study are available from the corresponding author upon request.

## Abbreviations

OVX: Ovariectomized
BMD: Mineral density
BV/TV: Trabecular bone volume
Tb.Th: Trabecular thickness
PRISMA: Preferred Reporting Items for Systematic Reviews and Meta-Analyses
STAIR: Stroke Therapy Academic Industry Roundtable
CI: Confidence interval
FDA: US Food and Drug Administration
DEXA: Dual energy x-ray absorptiometry
ROI: Regions of interest
ERs: Estrogen receptors
ERT: Estrogen replacement therapy
E2: 17-*β*-Estradiol
PTH: Parathyroid hormone
EX: Exercise
PBS: Phosphate-buffered saline
N: Nothing.
